# The Souss lagerstätte of the Anti-Atlas, Morocco: discovery of the first Cambrian fossil lagerstätte from Africa

**DOI:** 10.1038/s41598-021-82546-0

**Published:** 2021-02-04

**Authors:** Gerd Geyer, Ed Landing

**Affiliations:** 1 Lehrstuhl für Geodynamik und Geomaterialforschung, Institut für Geographie und Geologie, Bayerische Julius-Maximilians-Universität Würzburg, Würzburg, Germany; 2 New York State Museum, Albany, NY USA

**Keywords:** Palaeontology, Biodiversity, Sedimentology

## Abstract

Episodic low oxygenated conditions on the sea-floor are likely responsible for exceptional preservation of animal remains in the upper Amouslek Formation (lower Cambrian, Stage 3) on the northern slope of the western Anti-Atlas, Morocco. This stratigraphic interval has yielded trilobite, brachiopod, and hyolith fossils with preserved soft parts, including some of the oldest known trilobite guts. The “Souss fossil lagerstätte” (newly proposed designation) represents the first Cambrian fossil lagerstätte in Cambrian strata known from Africa and is one of the oldest trilobite-bearing fossil lagerstätten on Earth. Inter-regional correlation of the Souss fossil lagerstätte in West Gondwana suggests its development during an interval of high eustatic levels recorded by dark shales that occur in informal upper Cambrian Series 2 in Siberia, South China, and East Gondwana.

## Introduction

The Cambrian System is widely known for its surprisingly large number of fossil localities that show preserved soft tissues. These localities are commonly termed “fossil lagerstätte”^1^ and are a key for understanding one of the most important events in the history of life on Earth, the “Cambrian Explosion” or “Cambrian Radiation Event”^2,3^. During a comparatively brief period of only ca. five million years, multicellular organisms typical of the late Ediacaran^4^ were replaced by eumetazoans with modern anatomical characters that represent nearly all body plans seen in post-Cambrian bilaterian animals. The most important of these lagerstätten include the Burgess Shale of western Canada^5^ and later discovered assemblages from Chengjiang, South China^6^; Sirius Passet, North Greenland^7^; Emu Bay, South Australia^8^; and the Orsten localities of Sweden^9^. Additional Cambrian assemblages include the Qingjiang^10^, the Guanshan^11^ and the Kaili^12^ in South China, the Wheeler, Marjum and Weeks formations^13,14^ and the Spence Shale^13,15^ in the western United States, the Kinzers^16^ and Parker^17^ formations of the eastern United States, and the Kuonamka Formation^18^ of Siberia, among others. During the century that passed since Walcott’s discovery of the Burgess Shale, macroscopic, exceptionally preserved fossil assemblages have been discovered on all major Cambrian palaeocontinents, and more than 30 genera of non-biomineralizing organisms have been discovered from the Marjum Formation alone.

The Lower Palaeozoic of Morocco has attracted considerable attention due to the discovery of exceptionally preserved fossil assemblages in the Lower Ordovician Fezouata Shale of the Zagora area^19–21^ and Upper Ordovician of the Erfoud area^22^. In contrast, exceptional preservation in the Cambrian strata of Morocco was previously unknown except for a single xandarellid specimen found in the uppermost lower Cambrian strata in the High Atlas that reflects singular local obrutional preservation^23^ which did not regularly affect any of the other fossils frequently found in these strata. New data convincingly indicate the presence of a fossil lagerstätte in the Cambrian Stage 3 Amouslek Formation of the western Anti-Atlas, Morocco. This is the first recorded Cambrian fossil lagerstätte from Africa.

## Geological setting and regional distribution

**Figure 1:**
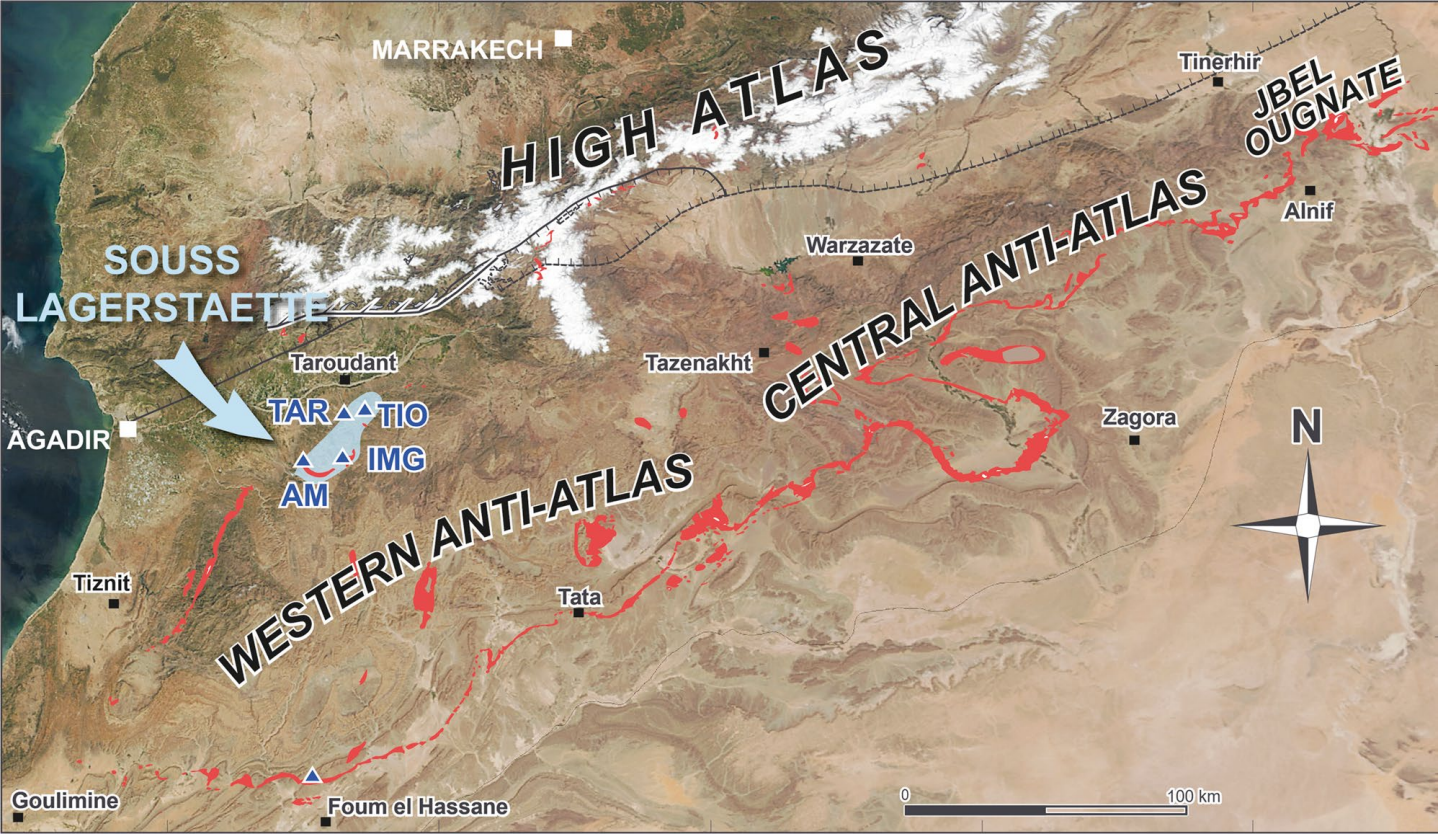
Satellite photo of the High Atlas and Anti-Atlas ranges in Morocco, with outcrop areas of Cambrian Series 2 strata (red) and localities yielding exceptionally preserved fossils within the Amouslek Formation (= Souss lagerstätte, blue). Abbreviations: AM, Amouslek; IMG, Imighzer; TAZ, Tazemmourt; TIO, Tiout. Satellite image modified from NASA-Modis picture GSFC_19032011 (temporarily available under http://commons.wikipedia.org and licensed under the Creative Commons Attribution 2.0 Generic license; modified by Adobe Illustrator CS5 and Adobe Photoshop CS5).

**Figure 2:**
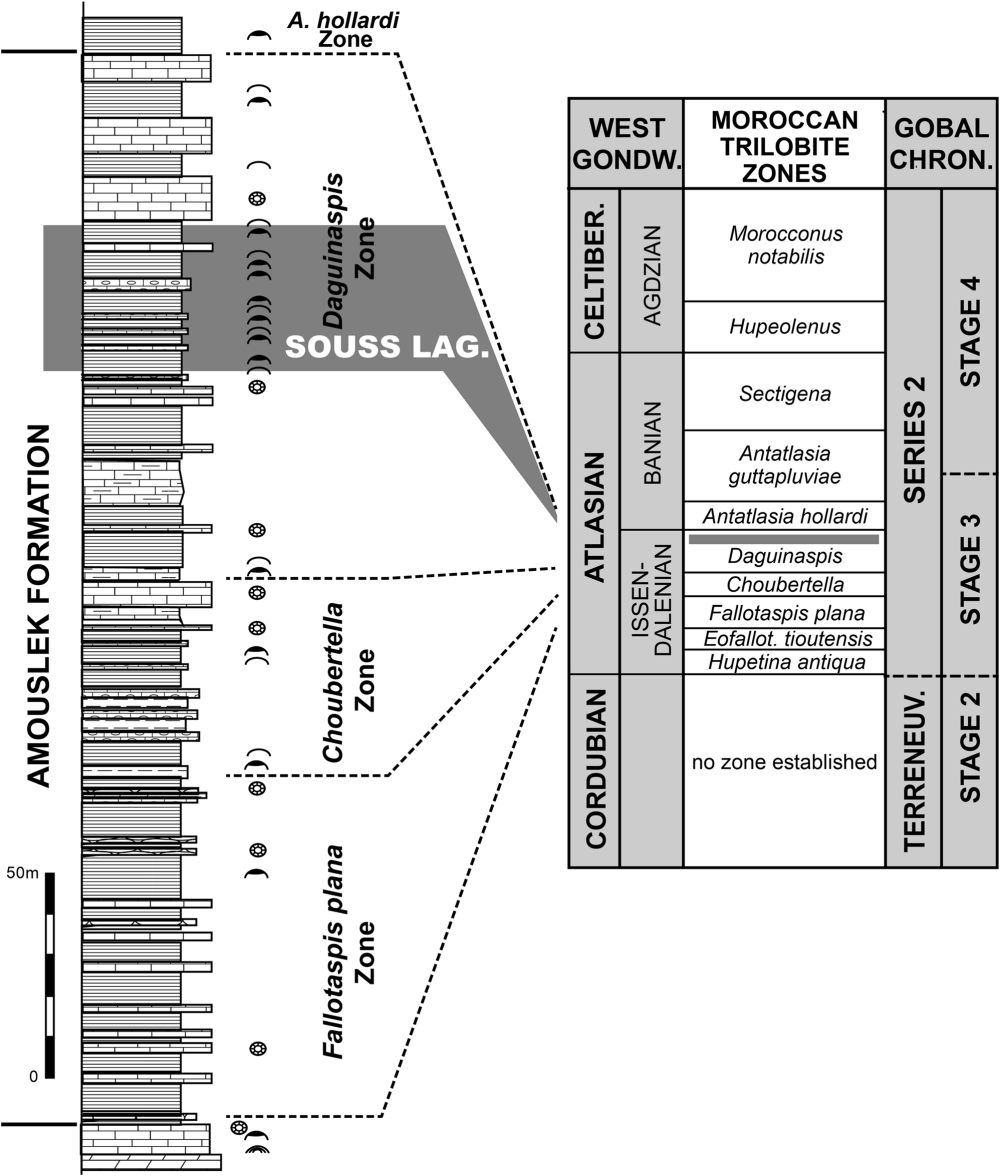
Stratigraphy of the Amouslek Formation at the northern slope of the western Anti-Atlas, illustrated by the Tazemmourt section (simplified, thickness of limestone beds exaggerated), with trilobite biozones, occurrences of trilobites, brachiopods and archaeocyaths and the position of the interval yielding exceptionally preserved fossils (shaded) in the early Cambrian. Abbreviations: *A*., *Antatlasia*; LAG, lagerstätte.

The “Souss fossil lagerstätte” (the name is based on the Oued Souss region between the Anti-Atlas and the High Atlas) is a hitherto unknown early Cambrian konservat lagerstätte in Morocco (Fig. 1). Similar to the distribution of the Chengjiang lagerstätte in South China, the Souss lagerstätte is not restricted to a single locality and stratum, but is represented by a more than ten metre-thick package which appears to be developed as stacked horizons with varying depositional conditions. The strata bearing fossils with unusual preservation locally amount to a possibly up to 40 m-thick interval of fairly unifacial rocks in the upper third of the Amouslek Formation^24^ (Fig. 2). The Amouslek Formation crops out along the northern slope of the western Anti-Atlas of southern Morocco, with exceptionally preserved fossils of the here described Souss Lagerstätte being found in an area between approximately 30° 10′ to 30° 25′ N and 9° 00′ to 8° 40′ W. The strata with these exceptionally preserved fossils belong to part of the *Daguinaspis* Zone^24^ which forms the upper part of the regional Issendalenian Stage corresponding approximately to the upper Lower Cambrian Stage 3. The formation is a succession of slightly argillaceous siltstones to fine-grained sandstones with only sparsely intercalated limestones (which elsewhere form frequent intercalations in the formation). These rocks are faintly laminated and have a light to bright yellow colour in outcrops due to weathering effects. These beds are macroscopically similar to other parts of the Amouslek Formation, but for the presence of exceptionally preserved fossils. Relatively fresh rock surfaces are medium grey in colour, and it is highly probable that this rock type, with limited evidence of bioturbation, represents short episodic low oxygenation events during deposition of the sediments.

## Preservation and diversity of fossils

The typical *Daguinaspis* Zone consists of fine-grained siliciclastic rocks in the western Anti-Atlas and contains a moderately diverse fauna dominated by fallotaspidid, bigotinid, abadiellid, dolerolenid, and despujolsiid (“resseropid”) trilobites^25–28^. A number of these trilobites are subject of professional fossil quarrying near the classical localities Tazemmourt and Amouslek, but only a very limited number of them show the unusual preservation described herein. In addition, linguloid, obolellid, botsfordiid and *Mickwitzia*-type brachiopods^29,30^, helcionelloid mollusks, hyoliths, and other, rare fossil taxa occur. Abundant archaeocyaths, microfossils (e.g., *Microdictyon*, *Hyolithellus*, *Allonnia*), and calcimicrobes are known from the thin and relatively sparse limestone beds. Potentially, soft part preservation can be expected from all these taxa, particularly the trilobites, brachiopods, hyoliths, and non-mineralizing organisms. Known to date are exceptional preservation in the trilobites *Fallotaspis bondoni*, *F. plana*, *Daguinaspis ambroggii*, *Perrector falloti*, *P. brevilimbatus*, *Marsaisia robauxi*, *M. devoillei*, *M. uncioculata*, the brachiopods *Brevipelta chouberti*, *Microschedia amphtrite* and an unidentified botsfordiid, a *Nevadotheca*-type hyolith and at least two additional unidentified hyolith species, a probably *Microdictyon*, *Hyolithellus*, and several unidentified organismal remains of uncertain systematic position. The succession is incompletely studied to date, with only few sections investigated in some detail. The best studied sections which yielded most of the unusually preserved trilobites are situated near the Tazemmourt village and belong to the classical fossil collecting sites of Morocco, with numerous small quarries at different levels of the upper Amouslek Formation. However, the three studied sections near Tazemmourt as well as the Imighzer and Tiout sections indicate substantial differences in the composition, occurrence and diversity of fossil assemblages, which suggests short-term and local fluctuations in environment and living conditions despite of superficially similar lithofacies of the shales.

**Figure 3:**
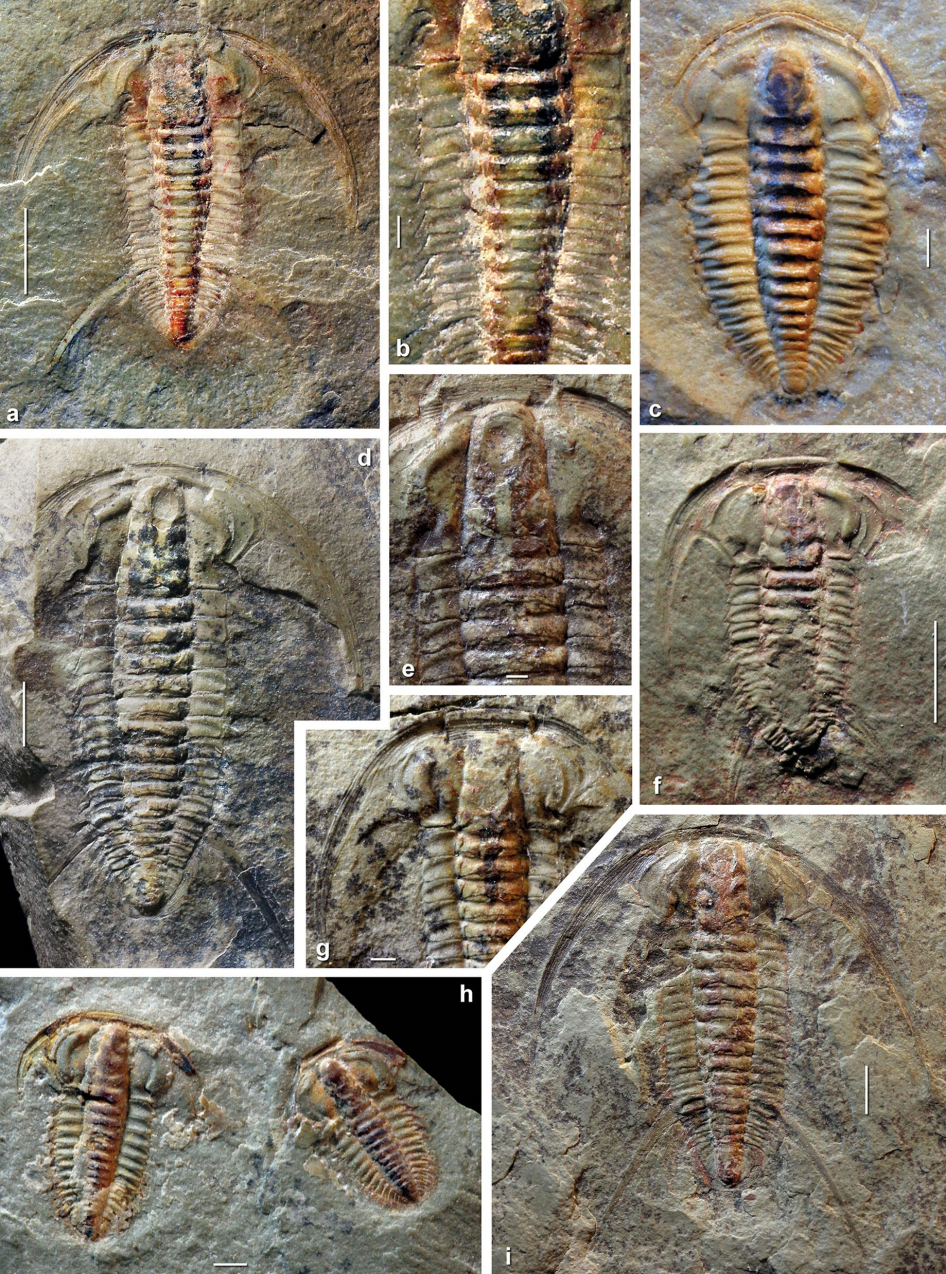
Examples of exceptional preservation of trilobites in the upper Amouslek Formation, *Daguinaspis* Zone, western Anti-Atlas, Morocco, specimens from the Tazemmourt section. (**a**, **b**, **d**, **e**, **i**), *Perrector falloti* (Hupé, 1953). (**a**, **b**) BOM 2529, internal mould of dorsal exoskeleton with series of paired digestive glands (dark stained, partly connected areas) under the axis of the cephalon and anterior thorax. (**d**) DEV 19.1F, internal mould of dorsal exoskeleton with series of paired digestive glands (blackish stained). (**e**) CGB Tr19a, internal mould of dorsal exoskeleton with impression of hypostome and centrally located alimentary canal running sagittally from the posterior margin of the hypostome to the fourth(?) thoracic segment. (**g**) CGB Tr26, internal mould of dorsal exoskeleton with mineralized, centrally located alimentary canal running from posterior part of the glabella to the third thoracic segment. (**i**) DEV C19.1B, internal mould of dorsal exoskeleton with partly preserved alimentary canal (reddish brown) and some paired digestive glands (dark stained) under the axis of the cephalon and most of the thorax. (**c**), *Daguinaspis ambroggii* Hupé, 1953, MMUW 2019E-001, internal mould of dorsal exoskeleton, first specimen of a fallotaspidid and olenelloid trilobite with club-shaped dark stains under the axial region and anterior thorax, interpreted as remains of paired midgut glands; note extended bilobate shape of the cephalic digestive glands below glabella and dark, iron stained small areas near thoracic spines on the left side. (**f**), *Perrector brevilimbatus* (Hupé, 1953), DEV C19.1P, internal mould of dorsal exoskeleton with subcentrally located alimentary canal underneath glabella and some paired digestive glands. (**h**), *Marsaisia* sp., DEV C19.1 Da, C19.1Db, two immature dorsal exoskeletons, largely exfoliated, partly covered with secondary calcite coat; exposed internal moulds show partly limonitized surroundings of the alimentary canal and digestive glands, with paired glands partly visible in the smaller specimens on the right side. Scale bars 5 mm in (**a**, **d**, **f**, **i)** 1 mm in (**b**, **c**, **e**, **g**, **h**).

**Figure 4:**
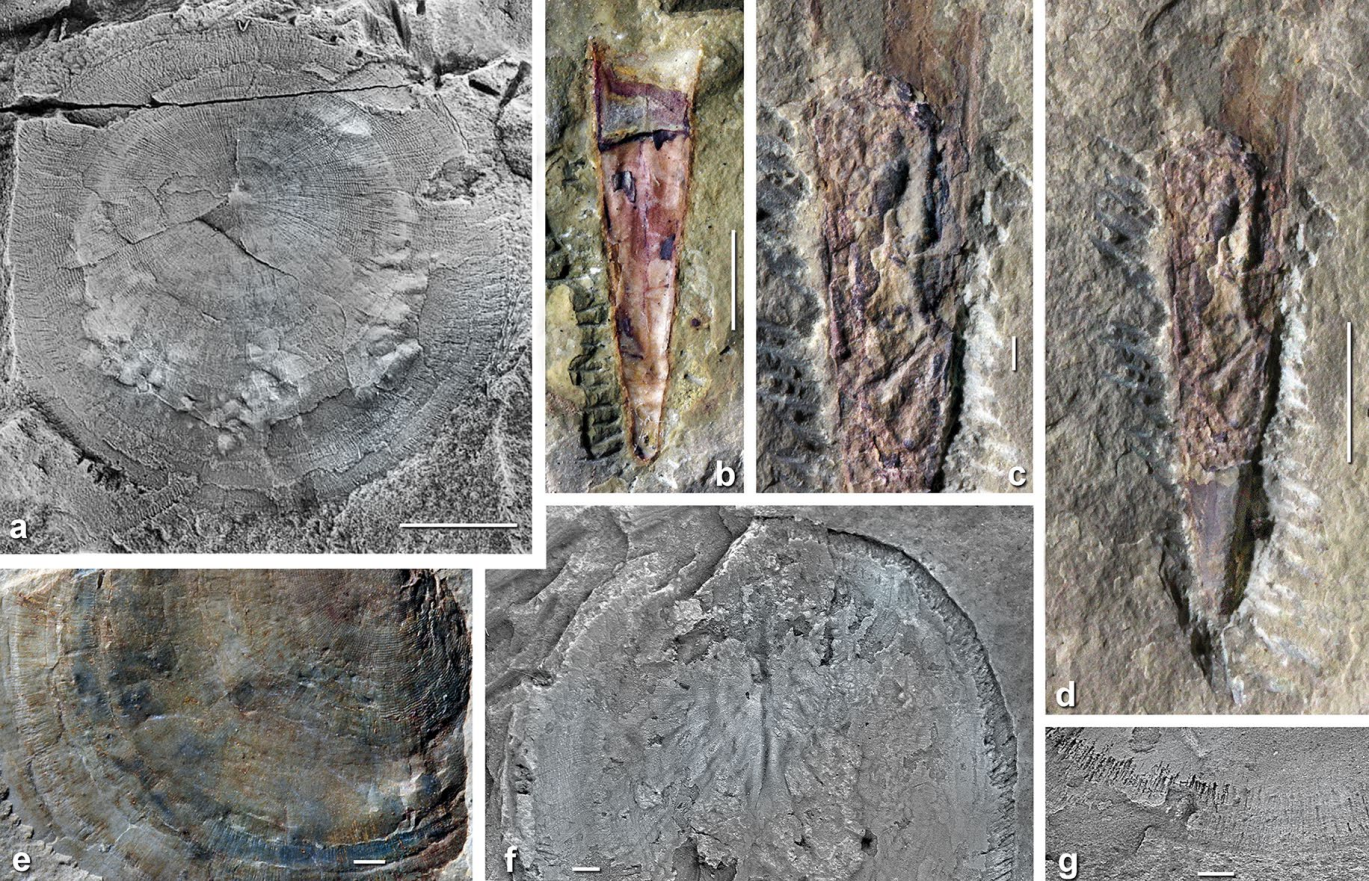
Examples of exceptional preservation in the upper Amouslek Formation, *Daguinaspis* Zone, western Anti-Atlas, Morocco, specimens from the Amouslek (**a**, **f**, **g**), Tazemmourt (**e**) and Tiout (**b**–**d**) sections. (**a**, **e**–**g**), *Microschedia amphitrite* Geyer^29^, mickwitziid brachiopod; (**a**) IGR 19998, visceral mass (presumably mostly parts the lophophore apparatus) pressed through the shell (visible as bulbous structures) and multiple, partly weakly mineralized shell layers (note punctate layer in the right half); (**e**) CGB 19c, partial valve with shell layers differently preserved and indeterminate internal organs indicated by concentric dark staining; (**f**, **h**) IGR 19988, slightly compressed shell with wrinkled surface partly exposing lower shell layers with granular tissue and partly dissolved edges with radially arranged crystallites; (**g**) IGR 19997, detail of shell showing partly edges with subradially arranged crystallites that indicate the arrangement of setae. (**b**–**d**), *Nevadotheca*? sp., hyolith; (**b**) MMUW 2019E-002, incomplete conch in “normal” preservation with shell secondarily limonitized, largely removed exposing calcitic infilling, but with external ornament (fine comarginal striation) engraved to the internal mould; (**c**, **d**) MMUW 2019E-003, incomplete, secondarily limonitized conch with apertural two-thirds largely broken; split middle part exposes poorly preserved parts of selectively mineralized soft tissues recognizable as discrete layers. Scale bars 5 mm in (**a**, **b**, **d**), 1 mm in (**c**, **e**, **f**, **g**).

Many fossils from this part of the Amouslek Formation preserve the remains of anatomical structures that are typically prone to post-mortem decay and therefore typically not preserved under more oxygenated conditions on the sea-floor. Examples of exceptional preservation include specimens of articulated trilobites of the species *Daguinaspis ambroggii* (Fig. 3c) and *Perrector falloti* (Fig. 3a,b,e) that show paired, metamerically-disposed, ovoid to club-shaped dark stains in the glabella and anterior part of the thoracic axis. These stains are interpreted to be incompletely preserved parts of the digestive tracts, particularly paired digestive glands and alimentary canal. Similar preservations can be seen in other specimens of *Perrector falloti* (Fig. 3d,g,i), in *Perrector brevilimbatus* (Fig. 3f) and in *Marsaisia* sp. (Fig. 3h). A different type of exceptional preservation of labile tissues is observed in specimens of the *Mickwitzia*-type brachiopod *Microschedia amphitrite*. Some of specimens of this species show remains of setae and dark colouration that correspond to parts of the visceral mass. The valves were demineralized during early diagenesis, and now show several preserved shell layers. In addition, the visceral mass was forced out from between the valves with compaction (Fig. 4a,e–g). Associated hyoliths (Fig. 4b–d) are variably preserved, with the shell material of the conch either demineralized or limonitized, but frequently with partial iron oxide moulds of the soft parts inside the conch. In addition, small fragments of arthropods without a mineralized dorsal carapace have been found on weathered bedding surfaces, along with a number of yet unidentified fragments that show labile tissues.

The limited number of isolated sclerites and the absence of sedimentary structures indicative of high-energy deposition would favour obrution as key mechanism to explain the preservation of these exceptional fossils. Taphonomic data such as very fine lamination and clayey lamina suggest restricted, but episodic wave-activity. Thus, the Souss lagerstätte must be classified as a konservat-lagerstätte sensu Seilacher^1^.

## Global relation

Intercontinental correlation of the strata indicates that the *Daguinaspis* Zone of the Moroccan Atlas ranges is partly coeval with the upper Atdabanian Stage of the Siberian Platform, the *Wutingaspis*–*Eoredlichia* Assemblage Zone of the lowermost Nangaoan Stage of South China, the “*Abadiella*” *huoi* Zone of South Australia^31^, and the dark mudstones of the lower *Calodiscus lobatus* Zone of NE Laurentia^34^ All four regions show relatively low oxygen levels in approximately coeval strata, with a pronounced development of black shales locally as in the Shujingtuo and Qiongzhusi formations of South China^32,33^. It is possible that coeval low-oxygen conditions could have developed synchronously and reflect high eustatic levels during a period of pronounced warm epeiric seas with varying and episidocally low levels of oxygen in solution^34^. Indications of such developments associated with low dissolved oxygen are provided by carbon isotope (δ^13^C_carb_) signatures. For the upper Amouslek Formation in the western Anti-Atlas, reconnaissance sampling has shown generally negative values^35^, but due to the limited number of carbonate beds in this stratigraphic interval, the significance of these values is highly uncertain. A negative peak is recorded from the coeval upper Atdabanian–lowest Botoman of Siberia^36^. By contrast, the Chengjiang, Qinjiang and Sirius Passet lagerstätten were related to the so-called CARE positive carbon isotope excursion^37^, which is commonly but probably erroneously regarded as a lower to middle Atdabanian equivalent^31^. However, biostratigraphic correlation clearly indicates that at least for the Chengjiang and Qinjiang lagerstätten are late Atdabanian (= late part of global Stage 3) equivalents (Fig. 2).

## Conclusions

This report features the first relatively abundant fossils with exceptional preservation from the Cambrian of Morocco (and Africa). The metazoan fossils now known with such exceptional preservation mostly belong to such characteristic invertebrate taxa as trilobites, brachiopods, and hyoliths, but the mode of preservation differs considerably among those groups. These fossils are found in a regionally and stratigraphically limited part of the upper Amouslek Formation (Cambrian Stage 3), where they occur through a thicker (up to ca. 40 m; Fig. 2) stratigraphic interval. The reason for the occurrence of exceptionally preserved fossils through this relatively thick interval reflects varying sea-water chemistry with short, episodic low oxygenated conditions on the sea-floor. The stratigraphic coincidence of this with other lower Cambrian fossil lagerstätte such as at Chengjiang, South China, raises the question of a possible global marine environmental fluctuation related to high eustatic sea levels and reduced oxygen in very warm epeiric seas^34^. Additional field work and analysis will further demonstrate the diversity of soft tissues preserved, their modes of preservation, and the environmental conditions that have facilitated their fossilization in this lower Cambrian interval of southern Morocco.

## Data Availability

The raw image files and all other data that support this study are available from the senior author.
